# How I Write Manuscripts for Peer-Reviewed Medical Journals

**DOI:** 10.7759/cureus.76452

**Published:** 2024-12-27

**Authors:** Khaled M Musallam

**Affiliations:** 1 Center for Research on Rare Blood Disorders (CR-RBD), Burjeel Medical City, Abu Dhabi, ARE; 2 Department of Public Health and Epidemiology, Khalifa University, Abu Dhabi, ARE; 3 Division of Hematology/Oncology, Department of Pediatrics, Weill Cornell Medicine, New York, USA

**Keywords:** medical journal, medical writing, publication, research, scientific communication

## Abstract

Writing manuscripts is an integral part of the research journey. Despite the availability of various guidelines to inform study reporting and manuscript preparation requirements by peer-reviewed medical journals, developing manuscripts that effectively communicate study findings or new knowledge requires a range of communication skills that evolve with successes and failures. In this manuscript, I feature some personal learnings and acquired habits in manuscript development and publication planning from my 15-year experience as a scholar, including insights on authorship matters, journal selection, manuscript type choices, medical writing of various data-driven and non-data-driven manuscript types, and handling revisions.

## Editorial

How should I start? Is my manuscript original 'enough'? Should I include these data or better not? Can I actually make these conclusions? What's taking the journal so long? These are some of the questions that keep authors awake at night. Writer's block and manuscript development dilemmas often lead to valuable data never seeing daylight or ending up in a researcher's procrastinatory to-do list, until they become too old to publish. Scientific writing is rarely a formal part of the education or training of scientists and physician scholars. Although journal and other guidelines on uniform requirements can give technical guidance, developing a manuscript for publication in a peer-reviewed journal requires an array of innate and acquired communication skills that often come with experience [1,2]. I always advise young scholars to try and avoid using generative artificial intelligence while writing their (first) manuscripts (even if permitted by the target journal). It takes away the 'fun' and 'reward' of the process and limits cumulative writing experience which I personally found to be a key factor for having more success in getting your research published.

I have had the privilege of leading manuscript development for over 200 publications of my own research projects and have frequently coached or supported the development of tens of publications for colleagues throughout my career, with roles and affiliations spanning various geographies including the Middle East, Europe/the United Kingdom, and the United States. I have also acted as a regular peer reviewer for over 30 medical journals and assumed several editorial roles over the past 15 years. It goes without saying that I have had my share of observations of successes and failures in getting research published and I herein reflect on my own experience with personal perspectives on manuscript development and publication, with the hope that this may be useful for aspiring scholars. My insights mostly relate to clinical research, especially observational studies, and non-data-driven manuscripts, since basic science and laboratory studies may have different reporting requirements.

Authorship: who's doing the heavy lifting?

Researcher roles on studies may not directly correlate with authorship roles on manuscripts. There are surely different ways of handling manuscript co-development by authors, but assigning a writing lead helps keep the process organized and timely. Nonetheless, all authors need to have made adequate contributions to both the study (for original research) and manuscript development, to qualify for authorship according to the International Committee of Medical Journal Editors (ICMJE) criteria adopted by peer-reviewed journals [3]. There is no formal guidance, however, on how to assign authorship order, a matter which can be sensitive and lead to disputes between authors. Although this is usually guided by 'idea ownership' and the extent of contribution to the study or manuscript write-up, it is often hard to quantify the weight of such contributions.

My first advice is to agree on the order early on so disputes can be addressed promptly. Authors are understandably eager to assume lead authorship positions (e.g., first author, last author, corresponding author) especially when these affect considerations for academic promotions. I have found that most authors usually settle for the most involved junior researcher to be the first author and the most senior to be the last/corresponding author. Unfortunately, I still commonly see junior researchers being denied lead authorship positions simply because of their 'junior' status, a behavior which should be strongly frowned upon and should not have a place in any academic setting nurturing the future generation of researchers.

There is always a possibility to designate equal contributions for lead authorship positions (e.g., first and second authors designated with an asterisk as co-first authors), but I often see this overused or adopted out of context. Group authorship can also be utilized for studies with a large number of authors, especially that contributors are now also listed in search engines and databases such as PubMed.

Getting support from a professional medical writer, with acknowledgment, may be needed especially for non-native English speakers or busy clinicians. Contract medical writing support is also often sought by the industry for sponsored clinical trials owing to the need for extensive internal tracking or documentation and quality control. This, however, may not be permitted for expert reviews, guidelines, and opinion pieces in many journals due to obvious conflicts. Irrespective of the writing party, authors still hold the ultimate responsibility for the manuscript and should never allow submission for publication without their full review and approval (part of the ICMJE authorship criteria). I have often seen manuscripts get retracted or corrected following publication, because some authors have only thoroughly read the manuscript following publication and disagreed with parts of the content or required alteration of their name spelling or affiliation.

I also always recommend that statisticians (whether part of the study team or contracted) get directly involved in manuscript writing for relevant parts, to ensure analyses methods and complex results are well presented and meet the expectations of peer reviewers who may happen to be statisticians.

Some authors could be anxious about financial disclosures (especially consultancy fees/honoraria from industry), assuming these may influence the chances of a manuscript getting accepted. Disclosures allow authors to be transparent with readers and do not necessarily mean that the work is considered automatically 'biased'. Multiple engagements with industry partners may in fact reflect expertise and thought leadership and may instill trust in the author's capabilities to conduct the study.

Publication planning and journal choice: the first door opener

Publication planning is essential for many reasons. Authors are often rushing/rushed to get their work published, which is understandable but not always achievable even with efficient editorial processing. Presenting data in congresses or preprints may help get results out there early on (with many congresses making those available in special journal supplements) and does not usually jeopardize chances for subsequent consideration by medical journals (may in fact work in favor).

Picking the 'right' journal for manuscript submission is key to ensure timely publication and avoid time wasted on editorial processing or review by journals that probably were not the wisest target choice to begin with, considering that manuscripts can only be submitted to one journal at a time. But how can one land on the right journal from the first attempt (or few, if I am to be realistic)? I usually use a combination of tactics. Indexing in major databases like PubMed is important to ensure wide visibility, and this is one criterion I hold dearly.

The second thing I look at is the journal impact factor. Although several entities today offer one, the Journal Citation Reports™ from Clarivate PLC remains the gold standard. In fact, I tend to use their master journal listings by category to identify journals for my discipline(s) of interest to begin with. The choices I make next rely on what I like to call 'the moment of truth'. We all want our manuscript to get published in high-impact factor journals, but we should be honest with ourselves about the true merit of the work. I never look at the absolute impact factor score per se, as the range may vary from discipline to discipline, but I look at quartiles. If I believe my work is among my finest, I confidently aim for a first-quartile journal, while I tend to aim lower with more conservative expectations of my work. Clinical trials tend to be favored by top-tier journals in respective categories or general medicine journals. That said, some new journals that did not get the chance to become PubMed indexed or get an impact factor yet may still attract my attention, especially if coming from a known publisher or published as a sister journal of one that I have high regards for. 

Although the title of the journal is revealing for its potential areas of interest, I still make the effort to look at the aims and scope section of the journal's website. I also browse the last few issues to get a sense of what the journal is focusing on lately. The best place to identify a target journal may in fact be my manuscript's reference list, and if I find myself frequently citing publications from a specific journal, it may be the one to consider for my submission. I surely also look at technical aspects of the journal's author guidelines like open access or publication fees, processing timelines, publication frequency, and, most importantly, the journal's accepted manuscript types, author number, word count, reference number, and figure/table/supplement allowances to ensure they meet my needs for an already developed manuscript or to guide me before even writing it.

I also find sending pre-submission queries to the journal very useful especially for review articles where a journal's interest in the topic is a key determinant for consideration as opposed to original research studies which may require more in-depth review by the journal to determine suitability. Some journals require pre-submission queries by default for authoritative manuscript types. Familiarity with certain journals and repeat successes may also build trust between authors and editors and encourage submission.

Regional interest also matters. I understand that colleagues in some regions always struggle when they get negative decision letters that advise authors to submit their work to 'regional journals' when these are practically limited or non-existent. It may be important to always highlight why your work applies to other regions or globally, when relevant. This is what I commonly do in cover letters accompanying my submissions; I make sure to spend some time to address the importance and relevance of my work to the journal's readership. I also give equal attention to my abstract and any other summary sections that the journal requires (key points, highlights, etc.) as these may be the first/only elements in your manuscript that are used for initial screening.

Lastly, journal transfer recommendations are now commonly adopted by journals from the same publisher, and I find these as a good option for quick transitions and avoiding extensive manuscript restyling for further consideration.

Original research manuscripts

Adhering to author guidelines during manuscript development is key to avoid delays in editorial processing and initial quality checks by the journal staff. Adopting international reporting guidelines such as the 'Standard Protocol Items: Recommendations for Interventional Trials' (SPIRIT) and 'CONsolidated Standards Of Reporting Randomised Trials' (CONSORT) for clinical trials [4-6], 'Strengthening the Reporting of Observational Studies in Epidemiology' (STROBE) for observational studies [7], 'Preferred Reporting Items for Systematic reviews and Meta-Analyses' (PRISMA) for systematic reviews and meta-analyses [8,9], and 'Standards for Reporting of Diagnostic Accuracy' (STRAD) for studies of diagnostic accuracy [10,11] is also highly recommended even if not mandated by the journal, as these allow standardization in reporting outcomes. Good resources for reporting guidelines (with explanations and links to the aforementioned ones) and manuscript preparation include the 'Enhancing the QUAlity and Transparency Of health Research' (EQUATOR) Network (https://www.equator-network.org/) and ICMJE (https://www.icmje.org/) websites. The 'Sex and Gender in Research' (SAGER) guidelines also provide recommendations on the reporting of sex and gender information in clinical studies [12]. Beyond these technical guidelines, authors would still need to charm their readers to gain their attention for the full length of the manuscript and ensure their key messages are delivered and retained.

For original research articles, most journals request the typical headings of introduction, methods, results, and discussion (IMRaD), commonly in that order although some may request the methods section to be presented towards the end. I have made it a habit to always start writing my methods and results sections before I develop the introduction and discussion, as I am not sure I would be ready to introduce a study before I am fully aware of how I am presenting what I did and found.

I also often choose my title upon completion of the whole manuscript and realizing the key messages it conveys. Although titles for non-data-driven manuscripts can be creative and catchy, original research manuscripts should aim for an informative title that reflects the design/aim or key result (some journals may prefer one over the other). In observational studies, I see many authors using titles that stress the fact that their study represents a center/country's experience. I get worried though that this may sway readers from other countries from the publication, seeing it as not relevant to their practice, even if the findings could be more widely applicable. Instead, I try to reflect key terms that differentiate and reflect my study's strength (e.g., the large sample size, the long follow-up duration, etc.). 

Beyond adherence to author guidelines and ensuring appropriate grammar and punctuation, I also try to make my submitted files (word docs, figures) look as 'neat' as possible, with consistent font types and sizes, homogenous line and paragraph spacing, and non-crowded tables and figures that have all elements clearly defined and explained. Messy-looking manuscripts may 'unconsciously' disturb reviewers while they try to focus on the scientific merit of the work.

Understanding journal guidelines when it comes to disclosures and forms is essential, and these may take a while to collect from co-authors. I would get on those as soon as I can when my target journal is identified. During manuscript submission, it is important to ensure that all information shared on the web portal is in keeping with what is in the manuscript. Authors should also take reviewer suggestions seriously, as it is not very uncommon for journals to rely on author recommendations.

Writing the Methods

The methods section needs to be a recipe of your work. Always consider readers as colleagues who may wish to replicate your work, so whatever you feel is useful towards that end needs to be included. Following a description of the study design, I detail all elements related to who was included in my study (e.g., eligibility criteria, disease definitions, procedures for participant identification/selection or record retrieval), when and where I did the study vs. when did the exposures/outcomes actually happen, and any other factors that allow the assessment of the generalizability of findings and potential sources of selection bias. When reviewing manuscripts, the first question I usually ask is: Is there a chance that the researchers included or left anyone out that they should not have? I then go on to describe my study variables and how they were defined and assessed.

The methods section should be a mirror image of the results section. Do not describe methods that are irrelevant to the results presented, and do not present results without a clear description of the methods that helped arrive at them. Tables or figures may be used for lengthy descriptions of study variables and procedures. Assessments that are standard and part of routine clinical care do not usually require detailed description, unless there is known variation in analytic methods. Even novel or experimental assays do not require lengthy descriptions (unless they are the core variable in the study), especially if I am able to cite other references that provide additional details on assessment and validation. Interventional studies would require an elaborate description of the study treatment and typical trial processes like randomization and stratification.

Ethical considerations are usually included in the methods, unless the journal requires these to go in another dedicated section. Using subheadings may be needed to better organize content. The past tense should always be used for the methods and results, and there is no harm in using the active voice (we conducted…).

The statistical analysis part is probably the only subheading that is always mandatory in the methods section. As mentioned earlier, the statistician who worked on data analyses should be involved in the write-up of this section. Although I usually perform my own analyses, I have made it somehow a habit to run my manuscript by colleagues who are trained statisticians for a nod of approval. I prefer to describe my statistical methods from the simplest to the more complex ones, but these can also be organized according to preset study endpoints. Data management aspects like special coding and handling of missing values should be transparent, since these are important for results to be appropriately interpreted.

Writing the Results

One common bad habit in writing the results section is the repetition of data between the text, tables, and figures. I usually structure my results section in the order of methods presented earlier, if feasible, or start with descriptive data followed by bivariate and then multivariate analyses or use any other logical flow (e.g., by endpoint hierarchy if applicable). Using subheadings is encouraged if it helps organize the flow of information.

I tend to spend a lot of time ensuring that most of my key results are reflected in tables and figures as these are often what I or my colleagues would use to present my data in congress presentations, so I am sort of making it easier for my study to be presented. Since I may choose to have my published figures printed in black and white (if I have no means to cover journal costs for color reproduction), I try to use colors/patterns in my illustrations that are still easily interpretable if printed in black and white. I then reflect on results that are statistically or clinically significant from those tables/figures in the text.

Supplementary appendices can be very useful to include data that may not otherwise 'fit' if journal word count or illustration allowances are restrictive. When reporting data, central tendency measures and point estimates should always be provided with dispersion or confidence intervals to allow the reader to properly interpret the full range of findings. Use and rounding of decimal points should follow logic; for instance, I do not see added value in reporting the mean age of a sample as 27.245 instead of 27.2 or even 27 years.

Writing the Introduction

I am personally not a big fan of long introductions. You really do not want to lose your reader's attention by the time they get to the core of your work, your methods and results. The introduction is all about building a strong yet concise case of why the study was done. I would avoid starting with cliché opening statements that describe the origins of a disease and would only include information essential to the reader based on the expected depth of knowledge for the journal's regular audience. For example, if I am submitting a paper on renal manifestations of thalassemia to a nephrology journal, I would spend more time introducing thalassemia than I would for a hematology journal. I would then summarize current knowledge and evidence gaps in one paragraph. The idea here is not necessarily to cite all available evidence but key highlights of it. I often rely on recent reviews to build my argument and end the introduction with a few lines stating my aims in the context of these evidence gaps.

I would avoid overcriticizing previous work if I am not certain that I can step up and address all limitations of previous studies. Identifying gaps in my own previous work, however, may be respected by the reader especially if they resonate with the aims of my current work. In trying to make my study sound unique, I do not put a lot of focus on it being the 'first' or 'largest' only, but more on how answering my research question can help build new knowledge relevant to someone, somewhere, and how my findings could impact current clinical practice.

Writing the Discussion

The discussion section does not also need to be overly long and does not necessarily need to reflect on every single result. More importantly, it should not be a repeat of the results, which the reader has just gone over. I would normally start with an opening paragraph that summarizes the main results in lay terms without referring to values extensively while highlighting key strengths. What follows next depends on the specific study, but I would usually spend two to three paragraphs comparing my data to the available literature and current practice, tackling individual themes.

Discussion sections are not meant to be a systematic review of the literature but rather a summary of previous work that is most relevant to the current study. I would surely not omit previous studies because they do not agree with my data. Self-citation is acceptable but should not overwhelm the discussion, although it is sometimes inevitable if you are among a handful of scholars who are interested in a specific research question (I am often guilty of that). I would then add my limitations paragraph which in my view should be a mandatory component of any original research manuscript. This does not necessarily need to be a confession list. I would certainly be transparent about all limitations of my study, but I also further expand on why this could still be 'ok' and does not necessarily jeopardize the quality of my work. Otherwise, I would indicate how these limitations could have swayed my results in one direction or the other. I also provide insights on how I or other researchers could do better next time.

The discussion should end with a brief concluding paragraph which again summarizes the main finding in one or two lines followed by some reflections on future research needs.

Writing the Abstract

I find abstracts as the hardest part to put together. After writing a couple of thousand words to detail your study, you now need to summarize it all in around 200-300 words. Irrespective of word count and required abstract structure, I would always spend most of my 'word quota' on summarizing the results rather than any other element. Abstracts may be the first (and only!) part of your manuscript that journal editors use to make an immediate assessment of the manuscript for further consideration and peer review. When selecting keywords, I try to use ones that are different from those used in the manuscript title to have more identifiers for search engines.

Manuscript Type

Original/research articles are not the only journal manuscript type that can include the results of research studies. Many journals also have brief/short reports or research letters as a potential home for your work. These usually come with stricter limitations of word count and illustration allowances, but they can still be the best manuscript type for your work. I write these using the same strategy and flow mentioned earlier while being as concise as I can to adhere to journal limits. I tend to go for these manuscript types when my results are focused in scope and my messages are brief, without the need for an extensive introduction or discussion of findings as results are self-explanatory. You may also have higher chances of getting your manuscript accepted than with original articles (no guarantees!), especially in high-impact factor journals.

Handling Revisions

In most instances, when journals send you a decision asking for a revision of your manuscript, it somehow means they believe that the reviewers' comments are addressable, although this may not always be the case since some suggestions require a repeat or change of study procedures which are not feasible. Otherwise, I always ensure to comply with the requirement of addressing the reviewers' comments 'point by point'. I do not always agree with or apply the reviewers' recommendations, who may be expressing personal views, but I am always thankful for their comments as they surely took away some valuable time from their busy agendas to referee my manuscript. I always try to make it easy for them to spot the alterations I made by clear reference to changes in the manuscript in my response letter or, even better, by copying the amended parts. I always make sure to end with a note that I am still open to further comments and I do not assume that my manuscript should now be accepted. If the manuscript is indeed accepted, I highly recommend reviewing journal galley proofs thoroughly (even with the usual tight turnaround timelines) to avoid needing to issue errata later on, especially with tables/figures which may have been completely redrawn by the publisher.

Non-data-driven manuscripts

Narrative and expert reviews can be invited or unsolicited. My focus in these is always to provide memorable and useful tables and figures so they can become a prime resource not only for citation but also for education. I try to avoid technical writing and listing of evidence like you would do in systematic reviews (which should be written as original articles) and ensure that I always weave in my opinion and insights alongside the evidence being reviewed. Reviews should tell a story and be original in the way you present the evidence and not necessarily in the evidence itself. I see many colleagues being discouraged from writing a review article simply because there is a recent one about a similar topic in the literature. I do not believe there should be any limitation on the number of reviews written about the same topic in the same period. First, different journals have different audiences, and when it comes to ensuring new knowledge about a disease or treatment is widely disseminated, the more reviews out there, the merrier. Second, different authors may present information with completely different interpretations, and such variation helps expose readers to different 'narratives' of the disease. Several other non-data-driven manuscript types are published by journals like viewpoints, perspectives, and commentaries, and I do not personally believe that these can only be written by top experts but should be open for any author who is able to skillfully explore various dimensions of science and clinical care.

Case reports

Case report sections are less frequently encountered in peer-reviewed journals. The rarity of featured cases probably means that they will not be extensively cited, which does not do the journal's impact factor good (calculated based on citations). These could present rare diagnoses, new driver mutations, off-label drug benefits, or previously unknown side effects. I always use clinical reason when deciding if my case is 'rare enough' and not only rely on a literature search, since it may be that similar cases were not considered interesting enough for publication by others. Writing case reports should be straightforward, including a brief introduction and discussion, while the focus should be on the description of the case. For the latter, I imagine myself describing the case to a fellow clinician, including all essential information but probably excluding basic clinical information that may not be as relevant from an academic point of view. Images are important to give 'flavor' to the case. In fact, when the figure (e.g., an interesting feature on MRI or a pathology slide) is all that is original and interesting, I would submit the case to an 'images' section in a journal. If the case is mainly interesting because of the challenge in arriving at a diagnosis or multidisciplinary team effort to treat the condition, there are other journal sections that may be more suitable for your case such as 'quizzes' and 'rounds', among others.

Table 1 and Table 2 summarize the perspectives shared in this manuscript.

**Table 1:** Perspectives on manuscript development and publication planning. Guidance stems from personal experience and can be considered when not in conflict with journal author guidelines. ICMJE: International Committee of Medical Journal Editors

Element	Guidance
Authorship	Assign a writing lead and writing roles
	Involve the statistician in the write-up where relevant
	Adhere to ICMJE authorship criteria
	Agree on authorship order early on
	Assign fair authorship positions to junior authors
	Consider (rationally) equal contribution designations
	Consider group authorship for large groups
	Ensure thorough final manuscript review and approval by all authors
	Be generous with disclosures
Publication planning and journal selection	Be patient and consider congress presentations and preprints if in a rush
	Factor journal indexing in journal choice
	Factor journal impact factor (choose quartile based on manuscript merit)
	Consider journal scope and current interests (browse latest issues)
	Get clues from your own reference list
	Check accepted manuscript types and word count/illustration allowances
	Consider pre-submission queries (especially for reviews)
	Develop familiarity with journals
	Consider regional interest and manuscript generalizability (reflected in the cover letter)
	Benefit from journal transfer options (for rejected manuscripts)
Original research manuscripts	Adhere to journal author guidelines
	Adhere to relevant reporting guidelines
	Develop the methods/results before the introduction/discussion
	Choose a differentiating yet informative title that captures broad interest
	Prepare submission files that are neat, proofed, and polished
	Collect required disclosures and forms early
	Provide thoughtful peer reviewer recommendations
Non-data-driven manuscripts	Reflect on the evidence presented in narrative/expert reviews
	Provide memorable tables/figures that can be used for presentation or education
	Distinguish flow and narrative from other reviews on the same topic
	Use a creative and catchy title
	Consider contributing to perspectives and opinion pieces
Case reports	Carefully assess originality (rarity)
	Focus on the case and describe it to a fellow clinician
	Consider the 'images' section in journals
	Consider other educational sections

**Table 2:** Further guidance on the development of original manuscripts.

Component	Guidance
Methods	Provide a recipe of your work
	Detail the who, when, and where (consider generalizability and selection bias)
	Detail study variables and procedures with just the right amount (possible cross-citation)
	Detail study treatment (interventional studies)
	Describe statistical methods (with statistician, simple to complex) and data management
	Mirror the results section
	Use past tense (also for results)
	Can use active voice
Results	Focus on tables and figures (ensure legible if printed in black and white)
	Avoid repetition between text, tables, and figures
	Use subheadings if necessary
	Start with descriptive, bivariate, and then multivariate analyses (or follow endpoint hierarchy)
	Provide dispersion for central tendencies and confidence intervals for point estimates
	Use logical rounding
Introduction	Avoid cliché openings
	Keep the target audience in mind
	Keep it brief and concise
	Provide a general overview of available evidence (cite reviews)
	Review unmet needs and evidence gaps (do not overcriticize)
	State study aims and reflect potential broad interest and implications
Discussion	Do not repeat results and values extensively
	Summarize key findings and strengths in lay words
	Compare to relevant literature and clinical practice by theme
	List limitations and discuss their implications (or lack of)
	Conclude with key message and future directions
Abstract	Give weight to results more than other parts
	Chose keywords not mentioned in the title
Manuscript type	Consider brief/short reports and research letters in addition to original/research articles
Revisions	Provide point-by-point responses
	Make it easy for reviewers to track your changes
	Feel free to respectfully disagree with reviewers
	Thoroughly review galley proofs for accepted manuscripts (especially tables and figures)

Writing manuscripts should consider both objective and subjective elements. At the end of the day, each author may have a different style and preference of how they would showcase their work to the medical community and interpret their data. However, ensuring data and messages are appropriately and effectively communicated is key to translate results and knowledge into applied evidence.
